# A Game of Russian Roulette for a Generalist Dinoflagellate Parasitoid: Host Susceptibility Is the Key to Success

**DOI:** 10.3389/fmicb.2016.00769

**Published:** 2016-05-24

**Authors:** Elisabet Alacid, Myung G. Park, Marta Turon, Katherina Petrou, Esther Garcés

**Affiliations:** ^1^Departament de Biologia Marina i Oceanografia, Institut de Ciències del Mar – Consejo Superior de Investigaciones CientíficasBarcelona, Spain; ^2^Laboratory of HAB Ecophysiology, Department of Oceanography, Chonnam National UniversityGwangju, South Korea; ^3^Departament d’Ecologia Aquàtica, Centre d’Estudis Avançats de Blanes, Consejo Superior de Investigaciones CientíficasBlanes, Spain; ^4^School of Life Sciences, University of Technology Sydney, SydneyNSW, Australia

**Keywords:** *Parvilucifera*, dinoflagellates, perkinsids, host–parasite interactions, specificity, prevalence

## Abstract

Marine microbial interactions involving eukaryotes and their parasites play an important role in shaping the structure of phytoplankton communities. These interactions may alter population densities of the main host, which in turn may have consequences for the other concurrent species. The effect generalist parasitoids exert on a community is strongly dependent on the degree of host specificity. *Parvilucifera sinerae* is a generalist parasitoid able to infect a wide range of dinoflagellates, including toxic-bloom-forming species. A density-dependent chemical cue has been identified as the trigger for the activation of the infective stage. Together these traits make *Parvilucifera*-dinoflagellate hosts a good model to investigate the degree of specificity of a generalist parasitoid, and the potential effects that it could have at the community level. Here, we present for the first time, the strategy by which a generalist dinoflagellate parasitoid seeks out its host and determine whether it exhibits host preferences, highlighting key factors in determining infection. Our results demonstrate that in its infective stage, *P. sinerae* is able to sense potential hosts, but does not actively select among them. Instead, the parasitoids contact the host at random, governed by the encounter probability rate and once encountered, the chance to penetrate inside the host cell and develop the infection strongly depends on the degree of host susceptibility. As such, their strategy for persistence is more of a game of Russian roulette, where the chance of survival is dependent on the susceptibility of the host. Our study identifies *P. sinerae* as a potential key player in community ecology, where in mixed dinoflagellate communities consisting of hosts that are highly susceptible to infection, parasitoid preferences may mediate coexistence between host species, reducing the dominance of the superior competitor. Alternatively, it may increase competition, leading to species exclusion. If, however, highly susceptible hosts are absent from the community, the parasitoid population could suffer a dilution effect maintaining a lower parasitoid density. Therefore, both host community structure and host susceptibility will determine infectivity in the field.

## Introduction

Historically, the role of parasitic protists in marine planktonic ecosystems has been largely neglected. New molecular tools have revealed that parasitism is a widespread interaction in aquatic microbial communities with a high diversity of unclassified parasites (Lefèvre et al., 2008; de Vargas et al., 2015) even in marine ecosystems not considered previously (Cleary and Durbin, 2016). There is increasing evidence that protist parasites may have a significant effect on plankton at the population, community, and ecosystem levels (Chambouvet et al., 2008; Lepère et al., 2008).

Parasite-mediated effects on their host populations are strongly dependent on parasitic specificity, i.e., the strength of the interactions between them (Anderson and May, 1981). Host species differ in their susceptibility to a certain parasite; therefore parasite transmission between species is often asymmetrical, where one host species might be highly infected resulting in a higher parasite load to the system (Woolhouse et al., 2001). In some host–parasite systems, generalist parasites infecting multiple host species possess traits to discriminate amongst host species (Krasnov et al., 2002; Goubault et al., 2004; Wang and Messing, 2004; Sears et al., 2012). Host abundance, species identity or host susceptibility are characteristics suggested to influence parasite preferences for choosing a certain host to infect in these systems, since these preferences are supposed to be adaptive strategies that maximize parasite fitness. Given that hosts can vary in their susceptibility to a certain parasite, and that host relative abundance in natural communities shift, parasite selection amongst host species is a very relevant question that has not yet been explored in great detail in parasitoid–phytoplankton systems.

Dinoflagellates are a dominant group of eukaryotic phytoplankton and an important component in marine ecosystem functioning, playing a key role in primary production and the marine food web (Margalef, 1978; Reynolds, 2006). Many dinoflagellate species can cause blooms and some of them produce potent toxins that cause negative impacts for human health, aquaculture and marine ecosystems (Zingone and Enevoldsen, 2000). Currently, three groups of zoosporic parasitoids with different phylogenetic origin are known to infect dinoflagellates, ‘*Amoebophrya ceratii’* complex (Syndiniales), *Parvilucifera* (Perkinsids) and *Dinomyces* (Chytrid), moreover, environmental molecular surveys have unveiled a high hidden diversity amongst these groups (Guillou et al., 2008; Chambouvet et al., 2014). The characteristics of these parasitoids are to kill their host, to have short generation times and to produce a huge amount of offspring following host infection (Coats and Park, 2002; Garcés et al., 2013a; Lepelletier et al., 2014a),thereby reducing the abundance of their hosts, potentially altering host population processes, such as competition, which in turn influence community composition.

Several studies have evaluated the range and specificity in host–parasitoid systems. In the case of the ‘*Amoebophrya ceratii’* complex, some clades are specialists (Chambouvet et al., 2008), whereas others have a broader host range (Coats and Park, 2002; Kim, 2006). However, in some generalist strains, after infecting a host, the offspring are unable to produce a second generation (Coats and Park, 2002). *Dinomyces* and *Parvilucifera* species (with the exception of *P. prorocentri*) have been described as generalist parasitoids, able to infect a wide range of hosts within dinoflagellates, including toxic species (Garcés et al., 2013a; Lepelletier et al., 2014a,b). In the case of *Parvilucifera* parasitoids, although a generalist in terms of the number of species they are able to infect, intra and inter-species variability still exists at the level of host susceptibility or infectivity (Figueroa et al., 2008; Råberg et al., 2014; Turon et al., 2015). The extent to which *Parvilucifera* parasitoids show preferences for certain hosts has not been fully investigated. Further research is required in order to understand the potential effects this parasitoid may have in marine microbial communities.

A system comprised of *Parvilucifera sinerae* and their dinoflagellate hosts provides a good model to address whether generalists *Parvilucifera* parasitoids exhibit preferences for the most susceptible hosts available, given that, (i) the reproductive success of the parasitoid depends on its ability to infect a host, (ii) it can infect a wide range of hosts from among dinoflagellates, and (iii) it uses chemical cues, such as dimethylsulfide, to detect host presence (Garcés et al., 2013b). As such, the objectives of the present work were to determine if *P. sinerae* shows preferences among possible dinoflagellate hosts, and evaluate whether the host susceptibility or the host dominance (in terms of abundance), are decisive factors when the parasitoid infects a host.

## Materials and Methods

### Host and Parasitoid Cultures

Experiments were conducted with host strains of several dinoflagellate taxa obtained from the culture collection of the Centro Oceanográfico (CCVIEO) in Vigo, Spain. Specifically, we used two strains belonging to Gonyaulacales: *Alexandrium minutum* (AMP4), and *Protoceratium reticulatum* (GC1AM); two strains belonging to Gymnodiniales: *Gymnodinium catenatum* (GC11V), and *Amphidinium carterae* (ACRN03); and two strains belonging to Peridiniales: *Scrippsiella trochoidea* (S3V), and *Heterocapsa niei* (VGO 623).

Cultures were maintained in 50 mL polystyrene tissue culture flasks filled with 20 mL of L1 medium (Guillard, 1995) without silica. The medium was prepared with filtered (0.2 μm), autoclaved seawater, adjusting the salinity to 31 by the addition of sterile MilliQ water. Cultures were grown at 20 ± 1°C with a photoperiod of 12:12 h (light:dark) cycle. Illumination was provided by fluorescent tubes with a photon irradiance of about 90 μmol photons m^-2^ s^-1^.

Stock parasitoid culture of *P. sinerae* (ICMB852) was propagated by transferring a 1 mL aliquot of mature sporangium every 6–7 days into an uninfected host stock culture of exponentially growing *A. minutum* strain AMP4 in sterile polystyrene six well-plates, each well with a growth area of 9.6 cm^2^ and a volume of 15.5 mL (BD Biosciences). These cultures were maintained under the same culture conditions mentioned above.

All experiments were conducted in triplicate using host cultures growing exponentially and recently formed sporangium of *P. sinerae* culture (strain ICMB 852). To obtain recently formed sporangia, 4 days after infection of an *A. minutum* (AMP4) culture, sporangia produced from the subsequent parasite generation were harvested for the inoculation of the experiments. Sporangia concentration was estimated by counting at least 300 mature sporangia (late sporocyte) using a Sedgewick-Rafter chamber. Zoospore concentration was estimated by multiplying the number of zoospores contained in a single sporangium (250 in the case of *A. minutum*; Garcés et al., 2013a) by the sporangia concentration. For the experiments, the volume added from the parasitoid mother culture was adjusted to obtain the final zoospore concentration required in each of the experiments.

### Parasitoid Generation Time and Transmission in the Different Host Populations

For each host species, triplicate 30 mL cultures at initial density of 1 × 10^4^ mL^-1^ were inoculated with recently formed sporangia at zoospore: host ratio of 1:60. We used this low parasitoid ratio to mimic the initial phase of an epidemic, avoiding killing the entire host population in the first generation, and then obtain two to three parasitoid generations in the same host population.

**FIGURE 1:**
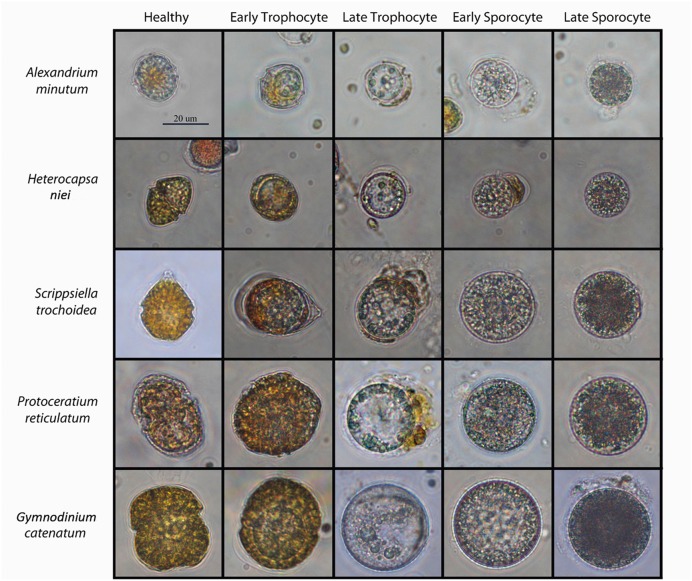
**Optical micrographs of the different life-cycle stages of *Parvilucifera sinerae* infecting five dinoflagellate hosts.** Scale bar = 20 μm.

Infected cultures were performed in 50 mL-polystyrene tissue culture flasks, and incubated under growth conditions (described above) for 14–16 days. This incubation time was required to observe at least two parasitoid generations depending on the host species that was infected. We took a 1 mL aliquot daily, preserved it with formaldehyde (1% final concentration) and the mature sporangia abundance were counted by inverted light microscopy (Leica–Leitz DMIRB) using a Sedgewick-Rafter chamber by scoring at least 300 sporangia, with the exception of the first generation, where the infections where very low. Mature sporangia (late sporocyte) of each host species can be seen in **Figure 1**.

Generation time was estimated by following the evolution of infected cells over time, which showed clear peaks associated with each parasitoid generation. We decomposed the evolution for each species into a sum of Gaussian peak shapes using an unconstrained non-linear optimization algorithm based on an iterative least-squares method, where the fraction of infected cells is the division of each individual Gaussian peak shape by the total number of infected cell at each time step. This fraction data allowed us to calculate the parasitoid generation time, following an adaptation of the methodology of Carpenter and Chang (1988) for the quantification of parasitoid generation time, by knowing the fraction of infected cells for each generation.

### Host Selection Experiment

Selection chambers were used to determine whether *Parvilucifera* zoospores demonstrated a putative behavioral attraction among three dinoflagellate species: the high-susceptible host *A. minutum*, the low-susceptible host *H. niei*, and the non-susceptible host *A. carterae*. Since, *A. carterae* is a dinoflagellate but not a potential host (*Parvilucifera* is not able to infect *Amphidinium*; see Table 2 of the study of Garcés et al., 2013a), we used this resistant species to know whether *Parvilucifera* zoospores are attracted to dinoflagellates in general, both those that can be infected (susceptible) as well as those not in their host range (resistant or non-susceptible). We also tested the attractiveness of two infochemicals, dimethylsulfide (DMS) and dimethylsulfoniopropionate (DMSP), which are related to dinoflagellate metabolism and were previously identified as chemical signals that activated the release of the zoospores from the dormant sporangium (Garcés et al., 2013b). Each selection chamber consisted of four 5 mL-syringes placed vertically, separated by 1 cm, into a 17-mL well volume (6-deep well plates, BioCoat^TM^) containing 15 mL of L1 medium (*n* = 9). In each of the nine wells, three of the syringes contained 1.5 mL of exudates from *A. minutum*, *H. niei*, and *A. carterae*, while the fourth syringe contained L1 medium (control). Exudates were prepared by filtering 5 mL of the host culture at 10^4^ cells mL^-1^ through 0.22-μm pore size Swinnex filters (Millipore) right before the experiment. Then, we added 1 mL of swimming zoospores at a concentration of 5 × 10^4^ in the center of the well and syringes remained dipped for 30 min. After this period, syringes were removed and the whole content inside the syringe was fixed with formaldehyde (1% final concentration). The number of zoospores that entered inside the syringe was estimated by counting at least 400 cells using a Sedgewick-Rafter chamber under light microscopy. To test whether the zoospores were attracted to specific chemicals cues, triplicate syringes containing lab-prepared DMS and DMSP at a concentration of 300 nM were placed inside a well filled with L1 medium and 5 × 10^4^ swimming zoospores. After 30 min, syringes were removed and zoospores were counted as above.

### Parasitoid Preferences for Host Species

Parasitoid preferences for infecting certain host species in an artificial mixed community of *A. minutum*, *S. trochoidea*, *P. reticulatum*, *H. niei*, and *G. catenatum* was tested in triplicate. The initial host concentration of each species was normalized by host cell biovolume in order to obtain a zoospore:host ratio of 1:1 taking into account the biovolume of 1.5 × 10^3^ *G catenatum* cells mL^-1^ which is the largest host. As the sizes of the host species used in this experiment vary, normalization by host cell biovolume avoids having different encounter probability rates between the parasitoid and the host. Infected cells of each species were counted during the first 3 days after parasitoid addition. We counted at least 300 cells as either infected or uninfected, identifying the infected ones of the whole artificial community by optical microscopy using a Sedgewick-Rafter chamber. Clear identification of the infected species was obtained, as infection is easily recognizable in the host species (**Figure 1** column 2: early trophocyte).

### Susceptibility of Host Species

Parasitoid prevalence in the five host species used in the preference experiment was determined as a function of inoculum size. For each experiment, sets of triplicate 50 mL-polystyrene tissue culture flasks containing 20 mL of host cells at initial density of 5 × 10^3^ mL^-1^ were inoculated with recently formed sporangia and incubated for 3–4 days under the same growth conditions as described above. Inoculum size of parasitoid for each set of triplicate vials was adjusted to give zoospores: host ratios of 1:1, 2:1, 5:1, 10:1, 20:1, 40:1, and 80:1. In two host species (*H. niei* and *G. catenatum*) the prevalence curve was not stabilized at ratio of 80:1, so we also inoculated both species with an inoculum size of 120:1 *ad hoc*. The time required to detect easily if the cell was infected or not was 3–5 days of incubation and that time was shorter than the time needed for the parasitoid to initiate a second round of infection (a second generation) according to the results obtained in the generation time experiment. After incubation, samples were preserved with formaldehyde (1% final concentration) and examined by inverted light microscopy (Leica–Leitz DMIRB) to estimate parasitoid prevalence. Parasitoid prevalence was calculated as a percentage of infected cells and was determined by scoring at least 300 cells per sample as infected (taking into account any of the infection stages) or uninfected (healthy) in a Sedgwick-Rafter chamber.

Data for each host species were fitted to a single two parameter exponential rise to maximum following the method of Coats and Park (2002). The equation for the curve fit was y = a (1 – e^-bx^), where a is the maximum infection level (I_max_) and b is α/I_max_. Alpha (α) represents the slope of the initial linear portion of the fitted curve and reflects the potential of zoospores to infect host cells. Alpha was estimated as I_max_^∗^b.

### Host Abundance Experiment

The effect of host abundance on parasitoid preferences was assessed in two systems; *System A* a mixed culture comprised of two species that were equally preferred in the preference experiment, *A. minutum* and *S. trochoidea*, and *System B*, a mixed culture containing a preferred host, *A. minutum* and a less preferred host, *H. niei*. For each system, we establish a set of triplicates in 50 mL-culture flasks of varying dominance with (i) the two hosts at the same initial host cell concentration (10^3^ cells mL^-1^); (ii) a mixed culture with *A. minutum* and *S. trochoidea* at 10^3^ cells mL^-1^ and at 10^4^ cells mL^-1^ initial cell concentration, respectively; (iii) a mixed culture with *A. minutum* and *S. trochoidea* at 10^4^ and at 10^3^ cells mL^-1^ initial cell concentration, respectively. The same set up was established for *System B;* the *A. minutum*/*H. niei* system. We inoculated 20 sporangia mL^-1^ of *P. sinerae* to each culture in order to obtain a 5:1 zoospore:host ratio matched to the less abundant host (10^3^ cells mL^-1^). By matching the zoospore ratio to the lowest density host we were able to minimize obscuring host preferences, as a higher number of zoospores could result in over-infection of both host populations, masking the true preference of the parasitoid. Prevalence in each host was determined during the first 4 days after parasitoid addition in *System A* and *System B*, by scoring at least 300 infected cells and identifying the species that was infected using a Sedgewick-Rafter chamber under light microscopy. All the infection stages were considered as infected when samples were counted, as shown in **Figure 1**, from the second to the last column (from early trophocyte to late sporocyte).

### Statistical Analyses

For the host selection experiment, to analyze whether *P. sinerae* zoospores were attracted by specific chemical cues (DMS and DMSP) and, if the parasitoid select among three different host species that differ in their susceptibility, we conducted a one-way analysis of similarity (ANOSIM). The analysis was performed on the number of zoospores that choose each treatment or each of the host species. ANOSIM is a multivariate non-parametric permutation test, analog to a one-way ANOVA (Clarke and Warwick, 1994). Prior to ANOSIM, similarity matrices were calculated by using the Bray-Curtis similarity coefficient. We used an α = 0.01 to test significance. In the case of significance, we conducted a *post hoc* test by multiple Pairwise Comparisons.

For the host preference experiment, to test if there were significant differences between species in the artificial community, we conducted the same statistical analyses as above, on the percentage of infected cells of each species at day three in the artificial community.

To test for significant differences in host susceptibility to the parasitic infection by *P. sinerae* we used two variables, the maximum infection level (I_max_) and the alpha value (α), which is the slope of the linear portion of the fitted curve. Prior to analysis data were transformed as log(X+1), because the two variables presented values that differed by one order of magnitude. Then, the same statistical analysis and *post hoc* test as above were performed. All the analyses were performed by using the statistical software PRIMER 6.1.2 (Clarke and Gorley, 2006).

## Results

### Parasitoid Generation Time in the Different Host Species

**FIGURE 2:**
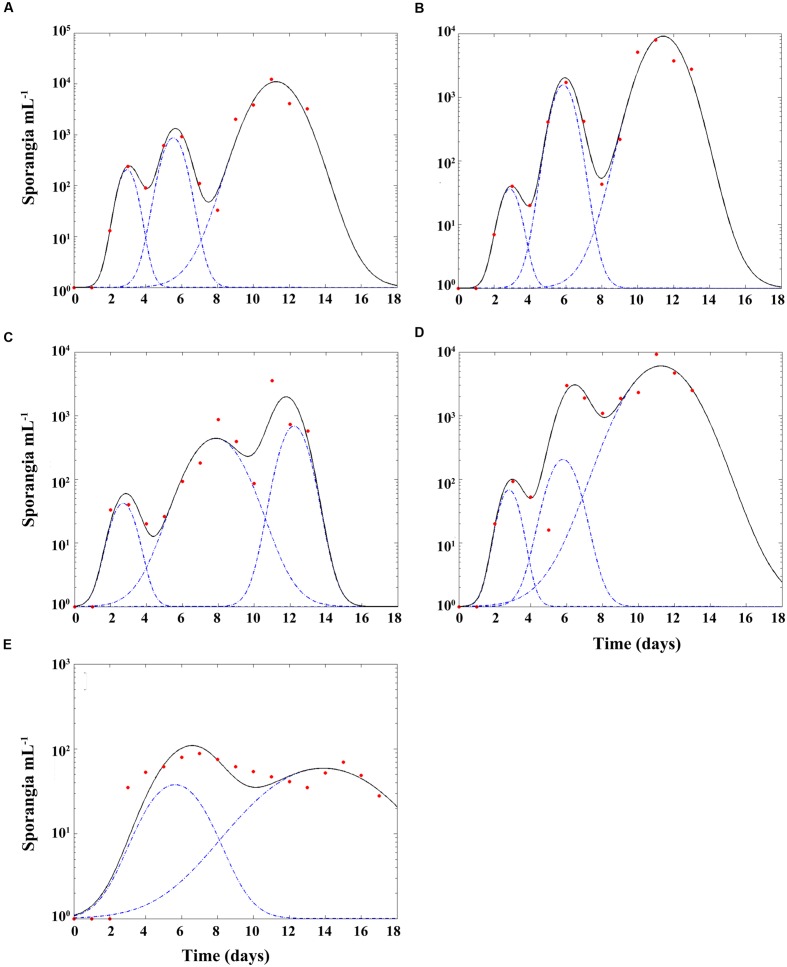
***Parvilucifera sinerae* generations in *Alexandrium minutum***(A)**, *Scrippsiella trochoidea***(B)**, *Heterocapsa niei***(C)**, *Protoceratium reticulatum***(D)**, and *Gymnodinium catenatum***(E)**.** Y-axis is the concentration of parasitoid sporangia (cells mL^-1^). X-axis is the time since parasitoid inoculation in days. Red dots are the observed concentration of sporangia. Black line is the fitted curve of sporangia concentration observed through the time. Blue dashed line is the peak of each generation predicted by the model. Note difference in y-axis scale in **(E)** which is two orders of magnitude lower.

Inoculation of *A. minutum*, *H. niei*, *S. trochoidea*, and *P. reticulatum* with zoospores at 1:60 ratio at a high initial host concentration of 10^4^ cells mL^-1^, produced an increased number of mature sporangia over the 16 days, showing three peaks corresponding to three generations of parasitoid life-cycle (**Figures 2A–D**). The same inoculation of *G. catenatum* resulted in a more gradual increase of the mature sporangia, showing only two peaks during the same time period (**Figure 2E**).

The estimated time for the first generation of *P. sinerae* was 62 and 137 h for the second generation in *A. minutum* (*r*^2^ = 0.98), being the species with the shortest generation time (**Figure 2A**). In the case of parasitoid infection in *Scrippsiella trochoidea* (*r*^2^ = 0.99; **Figure 2B**) and *P. reticulatum* (*r*^2^ = 0.92; **Figure 2D**) the averaged generation time was the same for both species, being 72 and 132 h for the first and the second generations, respectively. Infecting *H. niei* (*r*^2^ = 0.93; **Figure 2C**), the parasitoid showed a generation time of 108 and 154 h for the first and second generations, respectively. Finally, for *P. sinerae* infecting *G. catenatum* (*r*^2^ = 0.88; **Figure 2E**) we were only able to estimate the time for the first generation, because we observed two peaks, around 192 h. In all the species studied, the increase in the sporangia concentration through the different parasitoid generations was more than one order of magnitude between the successive peaks, with the exception of *G. catenatum*, where only low levels of infection were achieved in both generations.

### Host Selection

**FIGURE 3:**
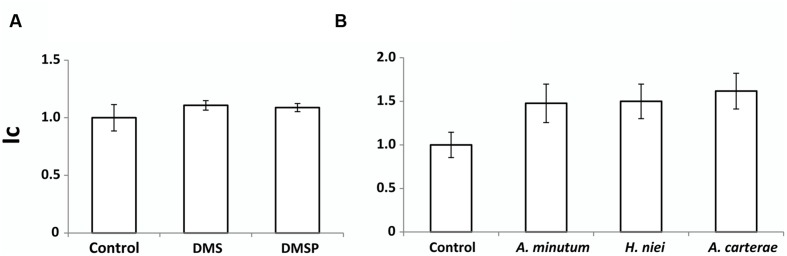
**Parasitoid zoospore chemotaxis for two chemical cues **(A)** and three dinoflagellate species **(B)**.** Ic is the chemotaxis index, defined as the proportion of zoospores that enter the syringe relative to the control (L1 medium). Data are expressed as mean ± SD.

The response of the zoospores to the info-chemicals DMS and DMSP was not different from that of the control (*p* = 0.23; **Figure 3A**). DMS, despite being involved in activating dormant zoospores inside the sporangium and acting as a chemical cue for high host abundance, did not play any role in host location. However, the response of zoospores to a signal from the three dinoflagellates species tested (**Figure 3B**) differed significantly from that of the control (L1 medium; *p* = 0.0001), suggesting the presence of a substance that is released by the living dinoflagellates which acts as a chemoattractant to the free-living parasitoids. Concerning host attractiveness through chemotaxis experiments, the pairwise comparisons between the different hosts, confirmed that zoospores did not present significant differences between host species (**Figure 3B**), indicating that the infective stage of *P. sinerae* does not select amongst its dinoflagellate hosts.

### Parasitoid Preference for Host Species

**FIGURE 4:**
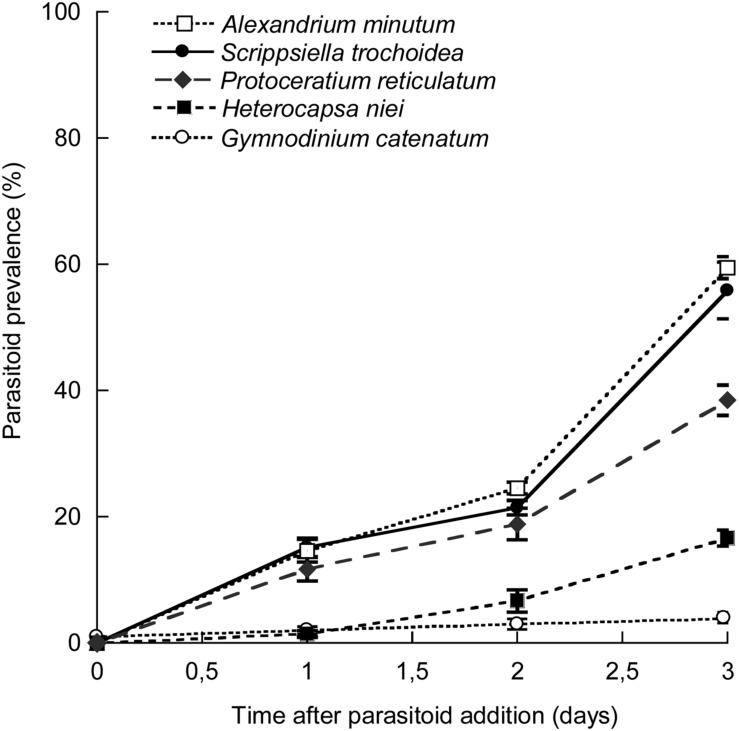
**Parasitoid prevalence (%) in each of the five host species mixed in an artificial community during the 3 days after parasitoid inoculation.** Data are expressed as mean ± SD.

Inoculation of *P. sinerae* in a mixed artificial dinoflagellate community revealed that the parasitoid preference for hosts significantly differed between host species (*p* = 0.0007; **Figure 4**). The parasitoid showed a gradient in the prevalence in the different hosts, showing the strongest preference for *A. minutum* and *S. trochoidea* species, reaching approximately 60% infection in both populations 3 days after parasitoid addition. The parasitoid showed no significant preference between these two species. The next most preferred species by *P. sinerae* was *P. reticulatum*, with 38% of its population infected, followed by *H. niei* (17%), and finally *G. catenatum*, which was hardly infected, showing infection prevalence in less than 3% of the population.

### Susceptibility of Host Species

**FIGURE 5:**
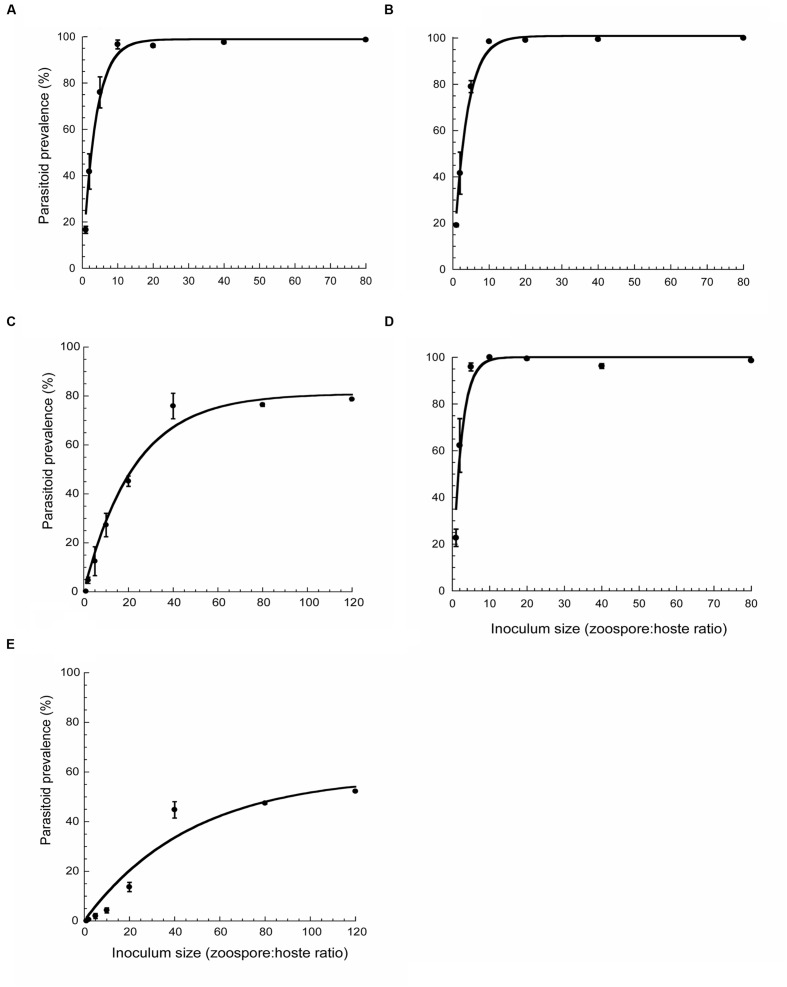
**Parasitoid prevalence as a function of inoculum size for *P. sinerae* infecting *A. minutum***(A)**, *S. trochoidea***(B)**, *H. niei***(C)**, *P. reticulatum***(D)**, and *G. catenatum***(E)**.** Host density was maintained at 5 × 10^3^ cells mL^-1^, with zoospore density varied to yield zoospore:host ratios of 1:1 to 120:1. Data are expressed as mean ± SD.

Parasitoid prevalence showed an exponential increase to a maximum relative to inoculum size in all five species tested (**Figure 5**). Estimates for maximum infection levels (I_max_) and initial slope of the fitted curves (α) were I_max_ = 98.2 ± 2.2; α = 27.4 ± 0.04 (*r*^2^ = 0.98) for *A. minutum*, I_max_ = 100.9 ± 1.91; α = 27.9 ± 0.04 (*r*^2^ = 0.99) for *S. trochoidea*, I_max_ = 100 ± 3.5; α = 28 ± 0.21 (*r*^2^ = 0.94) for *P. reticulatum*, I_max_ = 81 ± 3.5; α = 3.5 ± 0.01 (*r*^2^ = 0.94) for *H. niei*, and I_max_ = 58 ± 8.8; α = 0.98 ± 0.3 (*r*^2^ = 0.90) for *G. catenatum*. Host species varied significantly in their susceptibility to infection (*p* = 0.001) showing a gradient, with *A. minutum*, *S. trochoidea*, and *P. reticulatum* being the most susceptible (**Figures 5A,B,D**, respectively), followed by *H. niei*, and *G. catenatum* (**Figures 5C,E**, respectively). In the most susceptible species (*A. minutum*, *S. trochoidea*, and *P. reticulatum*) the maximum infection level was reached at 10:1 zoospore:host ratio where the whole dinoflagellate population was completely exterminated. In contrast, in the less susceptible species (*H. niei* and *G. catenatum*) the prevalence showed a more gradual increase to saturation (40:1 ratio) and failed to reach 100% infection levels, even at higher ratios (120:1).

### Effect of Host Abundance in Host Infection

**FIGURE 6:**
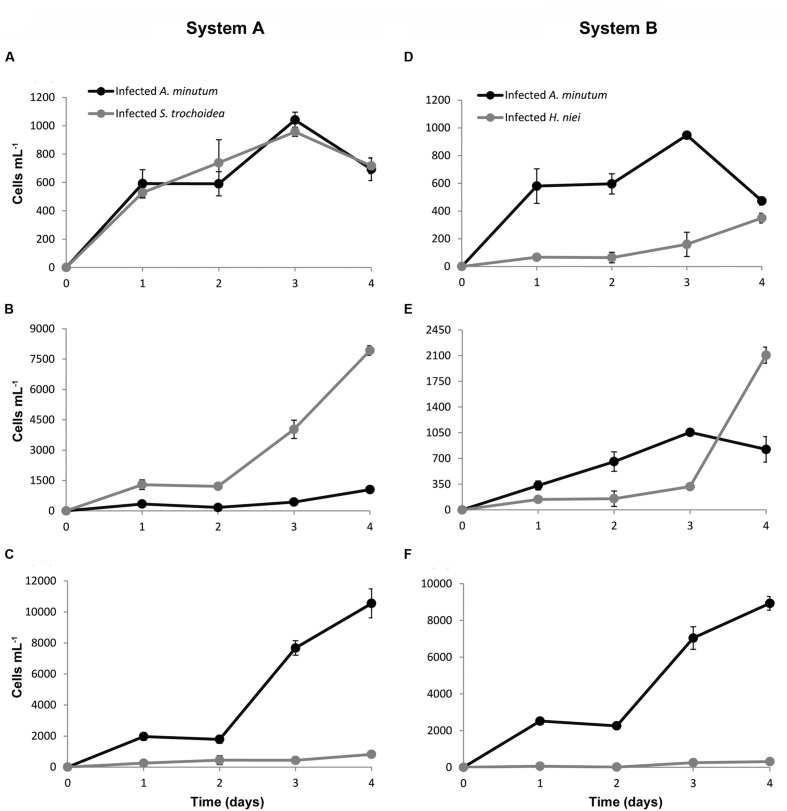
**Effect of host abundance in host infection in System A: a mixed culture of *A. minutum* and *S. trochoidea***(A–C)**, and in System B: a mixed culture of *A. minutum* and *H. niei***(D–F)**.** **(A,D)** Initial host density of both host was the same 10^3^ cells mL^-1^; **(B,E)** *S. trochoidea* and *H. niei* were at 10^4^ cells mL^-1^ and *A. minutum* was at 10^3^ cells mL^-1^. **(C,F)** *A. minutum* was at initial density of 10^4^ cells mL^-1^, and *S. trochoidea* and *H. niei* at 10^3^ cells mL^-1^. Data are expressed as mean ± SD.

The effect of host abundance in the choice of *P. sinerae* infection is highly dependent on host susceptibility (**Figure 6**). In the system comprised of equal host densities of two highly susceptible species, *A. minutum* and *S. trochoidea* (*System A*; **Figure 6A**), both species were infected without distinction. However, when the density of one of these species was higher than the other (**Figures 6B,C**), *P. sinerae* chose to infect the most abundant species in both experiments. In contrast, when the system was composed of one high-susceptible species (*A. minutum*) and one low-susceptible species (*H. niei*; *System B*), the parasitoid always reached higher infection in the one that is more susceptible, i.e., *A. minutum* (**Figures 6D–F**), independently of the initial density of the low-susceptible species. Nevertheless, an interesting effect was observed after the first generation took place in *System B* (**Figure 6E**), where after the parasitoid completed its first generation (day 3) killing the whole *A. minutum* population, the rapid increase in the parasitoid population allowed for high infection of the low-susceptible species *H. niei* (**Figure 6E**, day 4).

## Discussion

Parasitism is made up of many different strategies for infection, each one representing unique ecological interactions (Skovgaard, 2014). Understanding the relationship between parasitoids and hosts is crucial to know the role played by parasitoids, the impact that they can exert on a community and to quantify these processes for the modeling of natural phytoplankton communities.

### *Parvilucifera’s* Strategy of Seeking out a Host to Infect

In screening experiments*, P. sinerae* and the other species within the genera have been described as generalist parasitoids of dinoflagellates (Norén et al., 1999; Garcés et al., 2013a; Lepelletier et al., 2014b), however, the strategy of infection has never been studied. All *Parvilucifera* species complete their life-cycle in one individual host, which dies at the end of the infection. After reproducing, it produces many offspring inside a sporangium that remains dormant until the adequate signal. Garcés et al. (2013b) identified DMS as a density-dependent chemical cue for *P. sinerae* activation, where high concentrations of DMS communicate the presence of a high number of potential hosts in the marine environment. Upon activation, the zoospores abandon the sporangium in order to infect a new host. DMS is produced by several phytoplankton species, however, *Parvilucifera* are generalists so it follows that they may be activated by a general chemical cue. In this study, the chemotaxis experiment demonstrated that once outside the sporangium, the motile zoospores do not use the DMS/DMSP to locate a suitable host, but some other signal from living cells, which seems to be involved in host location (**Figure 3**). In a previous study involving an *Amoebophrya* parasite and the toxic *Karlodinium veneficum*, the authors found that high-toxin-producers were more infected than non-toxic strains (Bai et al., 2007). We did not measure the toxicity of the species tested; however, whether the parasitoid locates the host by a specific substance is an interesting question worthy of further investigation. Our results show that *Parvilucifera* does not select amongst potential dinoflagellate hosts tested in this study, instead, the parasitoid attacks all hosts encountered, regardless of species. In fact, the zoospores exhibit the same level of attraction to a high-susceptible, low-susceptible and a non-susceptible host. These data suggest that the infection strategy of *Parvilucifera* is more like a game of Russian roulette, where the zoospores seek out and contact a host at random, and it is only once the zoospores have encountered their host, that their fate is determined. Instead of choosing a host that will allow them to proliferate, successful infection is simply a game of chance and it is the hosts’ susceptibility that determines whether or not the parasitoid can attach and penetrate into the host cell to develop the infection.

### Parasitoid Preferences and Specificity

In the artificial mixed community, where the probability of encounter was the same for all dinoflagellate hosts used, we determined a preference for *Parvilucifera* to infect a certain species (**Figure 4**). A plausible hypothesis to test was that the parasitoid preferred to infect the largest host, as a strategy to increase parasitoid reproduction rate, since zoosporic parasitoids produce an amount of offspring proportional to host size, where the bigger the host biovolume, the more zoospores are produced (Garcés et al., 2013a). Certainly, the size of the host is significant, but, in terms of parasitoid transmission, parasitoid generation time in the different hosts and the number of hosts infected is also relevant. For instance, in this study the largest sporangium was obtained through infecting *G. catenatum*, but the total number of sporangia in two consecutive generations was orders of magnitude lower than in the other host species. Add to that the generation time, which was much longer, and the maximum population size of *P. sinerae* was much lower than the other more susceptible species. Moreover, in the preference experiment the greatest infection occurrence was reached in two species of different sizes, *A. minutum* and *S. trochoidea*, with a mean biovolume (*n* = 30 cells) of 1.6 × 10^3^ and 4 × 10^3^ μm^3^, respectively. As such, in the case of *Parvilucifera* parasitoids, the size of the host is not a determinant of host preference.

The *P. sinerae* strain used in this study was isolated from an *A. minutum* bloom, which often appears with *S. trochoidea* in the natural environment (Figueroa et al., 2008). Therefore, we cannot rule out the possibility that *Parvilucifera* shows an innate preference for a particular host species due to the result of historical sympatry., This would suggest that *P. sinerae* preferences are a result of host phylogeny, whereby the parasitoid easily infects more closely related dinoflagellates (Figueroa et al., 2008). The results of this study, where *P. sinerae* heavily infected *A. minutum* and *S. trochoidea* but not *G. catenatum* in the same extent (**Figures 4** and **5**), give weight to this idea of historical sympatry and are consistent with a study by Llaveria et al. (2010) on a natural population, in which *P. sinerae* heavily infected *A. minutum* and *S. trochoidea*, but not the more distantly related *Prorocentrum*. Similarly, results by Garcés et al. (2013a) support this idea, where *P. sinerae* was able to infect many species belonging to Gonyaulacales and Peridiniales, being less successful infecting Gymnodiniales and not able to infect any species belonging to Prorocentrales. Congruent results were obtained from *P. rostrata* and *P. infectans* (Lepelletier et al., 2014b), however, in the case of *P. prorocentri*, which is the most morphologically and phylogenetically distanced of the four *Parvilucifera* species described to date, it is the only *Parvilucifera* known to infect Prorocentrales (Leander and Hoppenrath, 2008).

We observed that *P. sinerae* prefers to infect *A. minutum* and *S. trochoidea* in a mixed community (**Figure 4**), which at the same time were the most susceptible species (**Figure 5**) showing (i) a high prevalence in the host populations, (ii) the zoospores being highly infective in these species (high α values), (iii) presenting shorter generation times, and (iv) producing denser parasitoid populations with each generation. So *P. sinerae* is well-adapted to its primary hosts maximizing parasitoid transmission, which could be a result of antagonistic coevolution. This refers to reciprocal evolution of host defense and parasitoid infectivity, which plays an important role in determining the outcome of infection. The study of Råberg et al. (2014) demonstrated that host susceptibility and parasitoid virulence in *P. sinerae*–*A. minutum* systems depends strongly on the combination of host and parasitoid genotypes involved. Also, these evolutionary processes could lead to intra-species phenotypic variability of several *P. sinerae* traits, such as host invasion and parasitoid transmission (zoospores success, infection rate and sporangia viability; Turon et al., 2015). Interestingly, *H. niei* and *G. catenatum* presented a higher resistance to parasitic infection, supporting higher zoospore load, which we had to increase to reach maximal levels of prevalence. Studies on parasite-induced defense reactions in dinoflagellate hosts to avoid infection are still scarce. Some hosts have evolved defenses by their capacity to produce cysts. Parasitoids alter or shift the community from planktonic life-forms to benthic, producing resistant cysts that avoid infection development (Toth et al., 2004; Chambouvet et al., 2011). Figueroa et al. (2010) found that parasitoid presence induced sexual recombination, where some phases of the life-cycle became infected but others did not, and promoting new host genotypes by genetic recombination that might be resistant to parasitic infection.

Our density-dependent experiments have shown that host abundance together with susceptibility, play an important role in parasitic infection (**Figure 6**), as *Parvilucifera* presents a frequency-dependent transmission. This is supported by the study of Johansson et al. (2006), which suggested that *P. infectans* distribution in the coast of Sweden is not only governed by the total dinoflagellate population but also the community dominance, which can significantly affect infectivity in the field. As our data show, in a situation of coexistence of two preferred competent species (those that propagate the parasitoid well, enabling its maintenance and spread), the host abundance is the determinant in the infection. The parasitoid will infect the most abundant species (**Figures 6B,C**), because the probability of an encounter with the dominant species is higher. In contrast, in a community dominated by two species with a different degree of susceptibility, for instance, *A. minutum* and *H. niei*, the key to parasitic infection is host susceptibility, where the parasitoid preferentially infects the most susceptible species rather than the most abundant one (**Figures 6D–F**). However, once the most susceptible host population has been infected during the first generation (**Figure 6E**), this newly increased parasitoid population allows *P. sinerae* to reach higher prevalence in the less susceptible host species during the second generation (see **Figure 6E**, day 4), as the level of infection depends on the parasitoid population size (**Figure 5C**).

### Potential Effects in the Community

The characteristics of zoosporic parasitoids are to kill their host, to have short generation times, to produce many progeny, and to exert top–down controls by reducing the size of their host populations, which in turn influence phytoplankton dynamics (Coats et al., 1996; Chambouvet et al., 2008; Velo-Suárez et al., 2013). Several authors have modeled the impact these parasites exert under a mono-specific dinoflagellate bloom situation (Montagnes et al., 2008), or in a three-host-species model (Salomon and Stolte, 2010) and the results obtained were similar to field studies. However, mono-specific dinoflagellate blooms happen only under very specific conditions, so most of the time phytoplankton communities are composed of a mixture of different species. Therefore, understanding the impact that generalist parasitoids infecting multiple dinoflagellate species could have on natural communities (i.e., *Parvilucifera* parasitoids) to incorporate in models is important, as it has the potential to completely change system’s dynamics (Dobson, 2004).

**FIGURE 7:**
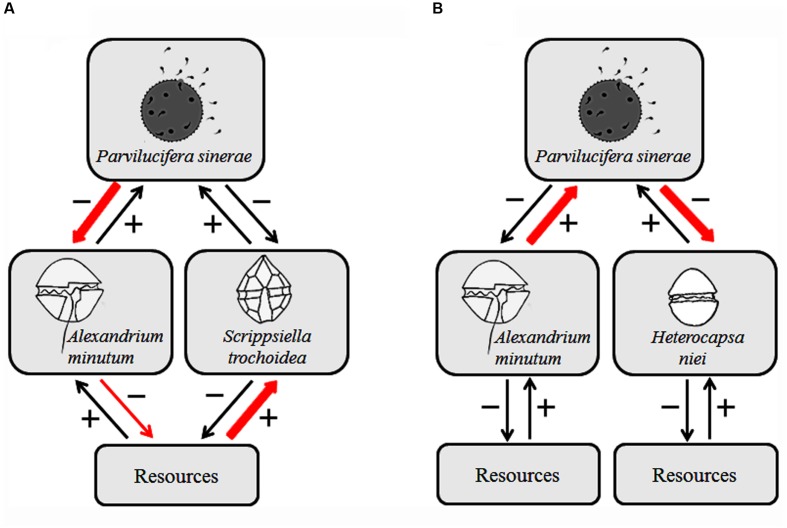
**Potential effects of *P. sinerae* (adapted from Figure 1 of Hatcher et al., 2012).** Arrows depict positive (+) and negative (–) direct effects (numerical effects) on population density resulting from the impact of a consumer or the resources; arrow thickness indicates strength of interaction; red arrows indicate key interactions, leading to the following patterns: **(A)** Parasitoid-mediated coexistence: regulation of a superior competitor by the parasitoid, i.e., *A. minutum*, enables *S. trochoidea*, less harmed by the parasitoid, to persist. **(B)** Apparent competition: higher densities of *A. minutum* host result in higher parasitoid population densities, which have a detrimental effect on *H. niei* host: thus, *A. minutum* acts as a reservoir of infection to *H. niei*.

The potential effects that a generalist parasite could have in the community are diverse, moreover, if it exhibits host preferences, the effects are potentially even more asymmetrical. *Parvilucifera*, as a generalist parasitoid, has a direct negative effect on the original host that they are infecting (*A. minutum*), which in turn may have an indirect effect, both positive and negative, on additional host populations and in those of non-host species. Our results suggest that, when competent hosts are present enabling a dense parasitoid population and good transmission, *Parvilucifera* plays an important role in shaping the structure of the community (**Figure 7**; Hatcher et al., 2012). In the first case (**Figure 7A**), *Parvilucifera* mediates coexistence of two competent species, *A. minutum* and *S. trochoidea*. The population of the most abundant species, or in other words the superior competitor, is regulated by parasitic infection, enabling the other, less-harmed species to persist. In this way *Parvilucifera* can enhance the coexistence of both species by reducing competitive advantage through preferential infection of the superior competitor. In an alternative situation where *Parvilucifera* is shared by two species with different susceptibility (**Figure 7B**), the most susceptible, *A. minutum*, acts as a reservoir of infection to *H. niei*. First, *Parvilucifera* infects *A. minutum*, its preferred host, where parasitoid transmission is highest, allowing the increase of parasitoid load. This in turn facilitates the infection of the less susceptible, but abundant *H. niei*, reaching higher levels of infection than would be attainable without the presence of the original host (**Figure 6E**). This situation can cause apparent competition, leading to species exclusion, as one host enhances parasitic infection in the other. In contrast to competitive and reservoir species, hosts that are inefficient propagators of *Parvilucifera*, like *G. catenatum*, can create a dilution effect, thereby lowering infection prevalence and reducing parasitoid population, but maintaining it in low concentrations until preferred hosts become dominant. In agreement to Lapchin (2002), in an unpredictable and changing environment, such as marine phytoplankton communities, *P. sinerae* biology and their infection plan makes for a successful strategy in the evolution of this species. *P. sinerae* is able to infect different species successfully, while having a higher fitness in a few of the hosts. This partial specialization allows the parasitoid to survive or maintain a small population when the most susceptible host becomes rare in the community.

Our results highlight the importance of understanding the mechanisms underlying specificity, which are presumably unique in each host–parasite system. The degree of specificity is very important when incorporating parasites into ecosystem models, especially for understanding how parasite prevalence and persistence impacts the marine microbial interactions, from the level of the community to the entire ecosystem.

## Author Contributions

EA: contributed to the design of all experiments included in the work, to the data aquisition, to the analysis and interpretation of the data and in drafting and writing the manuscript; MP: contributed to the design of generation time, host preferences, and host susceptibility experiments. He also contributed to the data aquisition, analysis and interpretation of these experiments. He revised the work critically; MT: contributed to the design of generation time, host preferences, and host susceptibility experiments. She contributed substantially to the data aquisition of these experiments and has revised critically the manuscript; KP: contributed in the design of host selection experiment and also in the aquisition, analysis, and interpretation of these data. She has substantially contributed to improve the manuscript for publication; EG: contributed in the design of all the experiments and in the interpretation of the results obtained. She also help in drafting the manuscript and revised for publication.

## Conflict of Interest Statement

The authors declare that the research was conducted in the absence of any commercial or financial relationships that could be construed as a potential conflict of interest.
